# Docoxahexaenoic Acid Induces Apoptosis of Pancreatic Cancer Cells by Suppressing Activation of STAT3 and NF-κB

**DOI:** 10.3390/nu10111621

**Published:** 2018-11-02

**Authors:** Mirae Park, Joo Weon Lim, Hyeyoung Kim

**Affiliations:** Department of Food and Nutrition, Brain Korea 21 PLUS Project, College of Human Ecology, Yonsei University, Seoul 03722, Korea; mirae_0114@naver.com (M.P.); jwlim11@yonsei.ac.kr (J.W.L.)

**Keywords:** apoptosis, docosahexaenoic acid, NF-κB, pancreatic cancer cells, STAT3

## Abstract

The ω3-polyunsaturated fatty acid docosahexenoic acid (DHA) is known to induce apoptosis of cancer cells. In this study, DHA was shown to reduce viability of pancreatic cancer cells (PANC-1) by inducing DNA fragmentation, activating caspase-3, and increasing the ratio of Bax/Bcl-2. To determine the DHA mechanism of action, the impact of DHA on the activation of the key signaling proteins epidermal growth factor receptor (EGFR), signal transducer and activator of transcription factor 3 (STAT3), nuclear transcription factor-κB (NF-κB), and IκBα in PANC-1 cells was probed. The observed DHA suppression of NF-κB DNA-binding activity was found to result from reduced IκBα phosphorylation. The observed DHA-induced suppression of STAT3 activation was found to be the result of suppressed EGFR activation, which derives from the inhibitory effect of DHA on the integrity of localization of EGFR to cell membrane lipid rafts. Since the activation of STAT3 and NF-κB mediates the expression of survival genes cyclin D1 and survivin, DHA induced apoptosis by suppressing the STAT3/NF-κB-cyclin D1/survivin axis. These results support the proposal that DHA-induced apoptosis of pancreatic cells occurs via disruption of key pro-cell survival signaling pathways. We suggest that the consumption of DHA-enriched foods could decrease the incidence of pancreatic cancer.

## 1. Introduction

Pancreatic cancer is the eighth leading cause of cancer death in South Korea. The five-year survival rate among pancreatic cancer patients is extremely low [1]. Because less than 20% of the cases are resectable, and the five-year survival rate after surgical resection is only 10–20% [2], a pressing need exists for the discovery of effective approaches to the treatment and prevention of pancreatic cancer.

As is the case with many other malignancies, constitutively activated signal transducers and activators of transcription factor 3 (STAT3) play a key role in pancreatic cancer [3]. The normal function of STAT3 is to regulate the proliferation, survival, and development of cells [4]. STAT3 is activated by several factors including interleukin-6 (IL-6), growth factor receptor, Janus-activated kinases (JAK), and Src family kinases. Once activated, STAT3 undergoes dimerization, nuclear translocation, and binding to its target genes. However, the aberrant activation of STAT3 contributes to oncogenesis by preventing apoptosis through the up-regulation of target genes such as cyclin D1 and survivin [5,6].

The epidermal growth factor receptor (EGFR), which activates STAT3, is reportedly overexpressed in human pancreatic cancer cells [7]. EGFR and its ligands, epidermal growth factor (EGF) and tumor growth factor-α (TGF-α), are coexpressed in the cells, which may contribute to the aggressiveness of pancreatic cancer by continuous EGFR signaling [8,9]. Ligand-bound EGFR autophosphorylates its cytoplasmic tyrosine residue, which can serve as a docking site for proteins like STAT3 that possess Src Homology 2 (SH2) domain [10]. In cancer cells, constitutive activation of the EGFR is common due to EGFR overexpression [11]. The EGFR becomes tyrosine phosphorylated and constitutively activated without a ligand when it is overexpressed.

Some of the genes regulated by STAT3 are also regulated by the protein complex known as nuclear transcription factor-κB (NF-κB) [12]. NF-κB is involved in pro-inflammatory and anti-apoptotic signaling pathways. In normal cells, NF-κB is located in the cytoplasm where it is maintained in an inactive state by the inhibitory protein IκBα. However, in cancer cells, NF-κB is not inhibited and, as a result, it functions to increase the expression of pro-survival genes such as cyclin D1 and survivin, resulting in uncontrolled cell growth [13,14]. STAT3 and NF-κB synergistically function to give cells the characteristics of cancer. One of the suggested mechanisms for this activity is that NF-κB, like RelA, can physiologically interact with STAT3 and their association can modify their transcriptional activity [15,16]. The other mechanism is cooperative binding of the two important transcriptional factors NF-κB and STAT3 at a subset of gene promoters to collaboratively induce expression of their target genes [17]. Through their functional interaction, NF-κB and STAT3 collaboratively promote tumor development via induction of pro-tumorigenic genes that affect angiogenesis and hypoxia, chemokines, and immunosuppressive cytokines [18,19,20].

Docosahexaenoic acid (DHA) is a ω3-polyunsaturated fatty acid found in cold oceanic fish oil. DHA has been reported to have protective effects against inflammation and against various cancers, including prostate, breast, and colon cancer [21,22]. DHA is known to induce the apoptosis of cancer cells by increasing the level of pro-apoptotic proteins while decreasing the level of anti-apoptotic proteins [22,23]. Several studies have suggested that DHA influences cell signaling by incorporation into plasma membrane lipid rafts thereby altering the localization of signaling receptors [24,25,26]. Lipid rafts are cell-membrane microdomains composed of cholesterol and sphingolipids, such as monosialotetrahexosylganglioside (GM1), that form a separate liquid-ordered phase in the liquid-disordered matrix of the cell membrane lipid bilayer [27,28]. Activation of EGFR, a ligand-independent surface receptor located in lipid rafts, is affected by the microenvironment [29]. Insight into the effect of DHA on cancer cells can be gained through determination of its impact on EGFR localization within the membrane lipid rafts.

The purpose of this study was to investigate the mechanism underlying DHA-induced apoptosis of the human pancreatic cancer cells, PANC-1. Specifically, the apoptotic effect of DHA on growth of pancreatic cancer cells was first assessed by determining the apoptotic indices (cell viability, DNA fragmentation, ratio of Bax/Bcl-2, and caspase-3 activation). Next, activation and interaction of STAT3 and EGFR, and the DNA-binding activities of NF-κB and STAT3 as well as the expression of their targeted cyclin D1 and survivin genes were investigated. Finally, we examined the effect of DHA on the integrity of and EGFR localization into cell membrane lipid rafts. The results of these experiments, reported below, support the conclusion that DHA-induced apoptosis of pancreatic cells occurs via disruption of key pro-cell survival signaling pathways.

## 2. Materials and Methods

### 2.1. Cell Line and Culture Condition

Human pancreatic cancer cells (PANC-1) were obtained from American Type Culture Collection (Rockville, MD, USA) and maintained in Dulbecco’s modified Eagle medium (DMEM) (Gibco, Grand Island, NY, USA) containing 10% fetal bovine serum and an antibiotic-antimycotic cocktail of 100 U/mL penicillin and 100 μg/mL streptomycin. The cells were incubated at 37 °C under a humidified atmosphere consisting of 95% air and 5% carbon dioxide. PANC-1 cells were grown to 80% confluency before use in experiments.

### 2.2. Experimental Protocol

PANC-1 cells were incubated with DHA (Sigma-Aldrich, St. Louis, MO, USA) dissolved in ethanol (final concentration of 50, 100 or 150 µM) for 4 h prior to the determination of phospho (p)-STAT3, STAT3, p-EGFR, and EGFR, the assays of NF-κB DNA-binding activity and STAT3 DNA-binding activity, and the measurement of lipid raft EGFR levels. The level of caveolin-1, which normally exists in the plasma membrane, was also determined in membrane fraction. Following a 24 h incubation period, DNA fragmentation was analyzed, the levels of pro-capase-3, cleaved caspase-3, Bcl-2, and Bax were measured, and the messenger RNA (mRNA) and protein expression levels of the genes encoding cyclin D1 and survivin were determined. Cells treated with 150 µM DHA for 4 h were employed in the immunoprecipitation of EGFR and STAT3 and the immunofluorescence assay for GM1. PANC-1 cells were also treated with AG1478 (Tyrophostin, EGFR phosphorylation inhibitor, AG Scientific, San Diego, CA, USA) dissolved in dimethyl sulfoxide (DMSO) (final concentration 10 µM) for 4 h (for determination of p-STAT-3 and STAT-3). Controls for the DHA-based experiments employed PANC-1 cells incubated with ethanol alone whereas those for the AG1478-based experiments used PANC-1 cells incubated with DMSO (less than 0.1%) alone.

For time course experiments, the cells were cultured with DHA (final concentration of 50, 100, or 150 µM) for 24 h and 48 h for determination of cell viability. The cells were treated with 150 µM DHA for 2, 4, and 6 h for measuring protein levels of p-STAT3, STAT3, p-EGFR, and EGFR.

### 2.3. Determination of Cell Viability

After 24 and 48 h of DHA treatment, cells were incubated for 3 h with 3-(4,5-dimethylthiazol-2-yl)-2,5-diphenyltetrazolium bromide (MTT) (thiazolyl blue; Sigma-Aldrich, St. Louis, MO, USA) dissolved in phosphate-buffered saline (PBS). The cells were lysed by mixing with 2-propanol in 0.1% HCl for 20 min using a shaker. The absorbances of the resulting mixtures were measured spectrophotometrically using a microplate reader (Molecular Devices, Sunnyvale, CA, USA).

### 2.4. DNA Fragmentation

DNA fragmentation was accessed by determining the amount of oligonucleosome-bound DNA contained in the cell extracts. The relative increase in nucleosomes in the cell extracts, determined at 405 nm, was expressed as an enrichment factor. The nucleosomes were quantified by using the sandwich ELISA assay (Cell Death Detection ELISA^PLUS^ kit; Roche, Indianapolis, IN, USA).

### 2.5. Real-Time PCR Analysis for Survivin and Cyclin D1

Total RNA was isolated by using TRI reagent (Molecular Research Center, Inc., Cincinnati, OH, USA). Total RNA was converted into complementary DNA (cDNA) by reverse transcription using a random hexamer and MuLV reverse transcriptase (Promega, Madison, WI, USA) and heating at 23 °C for 10 min, 37 °C for 60 min, and 95 °C for 5 min. The cDNA was used for real-time PCR with specific primers for survivin, cyclin D1, and β-actin. The sequences of the survivin primers used to produce the desired 498 bp PCR product were 5′-ATGGGTGCCCCGACGTT-3′ (forward primer) and 5′-TCAATCCATGGCAGCCAG-3′ (reverse primer). For cyclin D1 cDNA production, the desired 144 bp PCR product was obtained by using the forward primer 5′-ACAAACAGATCATCCGCAAACAC-3′ and reverse primer 5′-TGTTGGGGCTCCTCAG-GTTC-3′. For β-actin cDNA production, the desired 349 bp PCR product was obtained by using the forward primer 5′-ACCAACTGGGACGACATGGAG-3′ and reverse primer 5′-GTGAGGATCTTCATGAGGTAGTC-3′. For PCR amplification, the cDNA was amplified by 45 repeat denaturation cycles at 95 °C for 30 s, annealing at 55 °C for 30 s, and extension at 72 °C for 30 s. During the first cycle, the 95 °C step was extended to 3 min. The β-actin gene was amplified in the same reaction to serve as the reference gene.

### 2.6. Preparation of Cell Extracts

The cells were harvested by treatment with trypsin/ ethylenediaminetetraacetic acid (EDTA), followed by centrifugation at 1000× *g* for 5 min. The cell pellets were resuspended in lysis buffer containing 10 mM Tris pH 7.4, 15 mM NaCl, 1% NP-40, and protease inhibitor complex (Complete; Roche, Mannheim, Germany), and lysed by drawing the cells through a 1-mL syringe with several rapid strokes. The resulting mixture was incubated on ice for 30 min followed by centrifugation at 13,000× *g* for 15 min. The supernatants were collected and used as whole cell extracts. For preparation of nuclear extracts, the cells were extracted in buffer containing 10 mM (4-(2-hydroxyethyl)-1-piperazineethanesulfonic acid (HEPES) (pH 7.9), 10 mM KCl, 0.1 mM EDTA, 1.5 mM MgCl_2_, 0.05% nonylphenoxypolyethoxyethanol (NP)-40, 1 mM dithiothreitol (DTT), and 0.5 mM phenylmethylsulfonylfluoride (PMSF). The nuclear pellets were resuspended on ice in a nuclear extraction buffer containing 20 mM HEPES (pH 7.9), 420 mM NaCl, 0.1 mM EDTA, 1.5 mM MgCl_2_, 25% glycerol, 1 mM DTT, and 0.5 mM PMSF and then centrifuged. The supernatants were used as nuclear extracts. The protein concentration was determined by using the Bradford assay (Bio-Rad Laboratories, Hercules, CA, USA).

### 2.7. Western Blot Analysis for STAT3, p-STAT3, EGFR, p-EGFR, Bcl-2, Bax, IκBα, p-IκBα, Cyclin D1, Survivin, Caveolin-1, and Caspas-3

Aliquots from whole-cell extracts were loaded onto 7–14% sodium dodecyl sulfate (SDS) polyacrylamide gels (6–40 μg protein/lane) and separated by electrophoresis under reducing conditions. The proteins were transferred onto nitrocellulose membranes (Amersham, Inc., Arlington Heights, IL, USA) by electroblotting. The transfer of protein was verified using reversible staining with Ponceau S. The membranes were blocked using 3% non-fat dry milk in TBS-T (Tris-buffered saline and 0.2% Tween 20). The proteins were detected using antibodies for p-STAT3 (#9131, Cell Signaling Technology, Danvers, MA, USA), STAT3 (06-596, Upstate Biotechnology, Lake Placid, NY, USA), p-EGFR (sc-81488, Santa Cruz Biotechnology, Dallas, TX, USA), EGFR (SC-373746, Santa Cruz Biotechnology), Bcl-2 (sc-492, Santa Cruz Biotechnology), Bax (sc-526, Santa Cruz Biotechnology), IκBα (sc-371, Santa Cruz Biotechnology), p-IκBα (#2859, Cell Signaling Technology), cyclin D1 (sc8396, Santa Cruz Biotechnology), survivin (sc-10811, Santa Cruz Biotechnology), caveolin-1 (SC-53564, Santa Cruz Biotechnology), caspase-3 (#9662S, Cell Signaling Technology), and actin (sc-1615, Santa Cruz Biotechnology) in TBS-T solution containing 3% dry milk, and incubation overnight at 4 °C. After washing with TBS-T, the primary antibodies were detected using horseradish peroxidase-conjugated secondary antibodies (anti-mouse, anti-rabbit, anti-goat), and visualized by exposure to BioMax MR film (Kodak, Rochester, NY, USA) using the enhanced chemiluminescence detection system (Santa Cruz Biotechnology). Actin served as a loading control. The ratio of Bax/Bcl-2 was determined from the protein-band densities of Bax and Bcl-2. The values are expressed as ±S.E.M. of four different experiments.

### 2.8. Immunoprecipitation of EGFR and STAT3

Cells were extracted with lysis buffer (1% Triton x-100, 0.1% SDS, 0.1 M NaCl, 0.01 M NaPO_4_, 1 mM PMSF, 2 μg/mL aprotinin, 0.2 mM Na_3_VO_4_, 50 mM NaF, 2 mM EDTA, 0.5% deoxycholate) as previously described [19]. The extract was incubated with beads overnight at 4 °C. After washing the beads four times, they were boiled with 2× loading buffer containing mercaptoethanol and SDS for 10 min to separate and denature the proteins, followed by SDS-PAGE analysis.

### 2.9. Luciferase Reporter Gene Assay for NF-κB Activity

Cells were transfected by 16 h incubation of the NF-κB reporter plasmid, the pRL-TK vector (containing the herpes simplex virus thymidine kinase (HSV-TK) promoter to provide *Renilla* luciferase expression), and the FuGene HD transfection reagent (Promega, Madison, WI, USA). Following 4 h of DHA treatment, the cells were lysed with passive lysis buffer (Promega). The activities of firefly luciferase and *Renilla* luciferase were measured by dual luciferase assay according to the manufacturer’s instruction.

### 2.10. Electrophoretic Mobility Shift Assay (EMSA) for NF-κB and STAT3

The NF-κB gel shift oligonucleotide (5′-ACTTGAGGGGACTTTCCCAGGGC-3′) and the STAT3 gel shift oligonucleotide (5′-GATCCTTCTGGGAATTCCTAGATC-3′) were radiolabeled using [^32^P]-deoxyadenosine triphosphate (dATP) (Amersham Biosciences, Piscataway, NJ, USA) and T4 polynucleotide kinase (GIBCO, Grand Island, NY, USA). The radiolabeled oligonucleotide was separated from unconsumed [^32^P]-dATP using a Bio-Rad purification column (Bio-Rad Laboratories) eluted with Tris-EDTA buffer. Nuclear extracts of the cells were incubated with the [^32^P]-labeled oligonucleotide in buffer containing 12% glycerol, 12 mM HEPES (pH 7.9), 1 mM EDTA, 1 mM DTT, 25 mM KCl, 5 mM MgCl_2_, 0.04 μg/mL poly[d(I-C)] at room temperature for 30 min. The samples were subjected to electrophoretic separation at 4 °C on a nondenaturing, 5% acrylamide gel. The gel was dried at 80 °C for 2 h after which it was exposed at −80 °C to a radiography film using intensifying screens.

### 2.11. Immunofluorescence Staining for GM1 in Lipid Rafts

For detection of lipid rafts, cells seeded on a slide glass were treated with 150 µM DHA for 4 h. GM1, a marker for lipid rafts, was stained with Alexa-fluor 555-conjugated cholera toxin subunit B (CTB-555) and detected with confocal microscope. Briefly, 15 min prior to fixation, CTB-555 (C-34776, Life technologies, Carlsbad, CA, USA) was added. The cells were fixed with cold methanol and then blocked for 1 h using a blocking solution (1% bovine serum albumin (BSA), 0.1% gelatin) before incubating with GM1 antibody (CAS 71012-19-6, Santa Cruz Biotechnology) for 1 h. After washing with PBS, the cells were reacted with fluorescein isothiocyanate (FITC)-conjugated mouse anti-goat IgG antibody (Santa Cruz Biotechnology) for 1 h and then washed. Next, 5 μg/mL of 4′,6-diamidino-2-phenylindole (DAPI) in blocking solution was added (for the purpose of nucleus detection) before subjecting the cells to laser scanning confocal microscopy (Zeiss LSM510, Carl Zeiss AG Corporate, Oberkochen, Germany).

### 2.12. Isolation of Lipid Raft Fractions and Determination of Calveolin-1 and EGFR in Membrane Fractions

Detergent-resistant membranes containing lipid rafts were isolated as previously described [30]. Briefly, cells were lysed on ice with TNE-P buffer (25 mM Tris-Cl, 150 mM NaCl, 5 mM EDTA, 0.2 mM phenylmethylsulfonyl fluoride (PMSF), 2 mM Na_3_VO_4_) containing 1% Triton X-100. After 10 min of microcentrifugation, the supernatant was saved as the soluble fraction. The pellet was resuspended with TNE-P buffer containing 60 mM octyl glucoside to solubilize the detergent-resistant membranes. After 20 min of microcentrifugation, the supernatant was saved as the insoluble fraction. EGFR and caveolin-1 levels in the respective soluble and insoluble fractions were determined by using Western blotting as described above.

### 2.13. Statistical Analysis

One-way ANOVA, followed by Newman-Keul’s post hoc tests, were used for statistical analysis. All data are reported as the mean ± S.E. of four independent experiments. A *p*-value of 0.05 or less was considered statistically significant.

## 3. Results

### 3.1. DHA Induces Apoptosis in PANC-1 Cells

**Figure 1:**
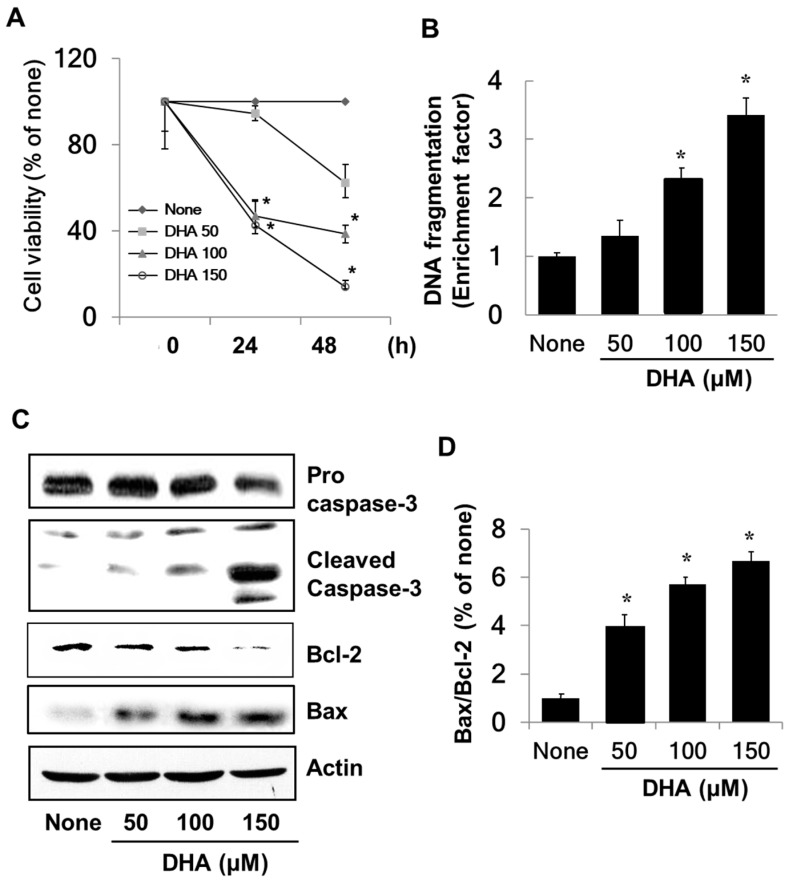
The effect of docosahexenoic acid (DHA) on cell viability, DNA fragmentation, protein levels of caspase-3, Bcl-2, and Bax, and the Bax/Bcl-2 ratio in pancreatic cancer cells (PANC-1) cells. Cells were treated with the indicated concentrations of DHA for 24 h and 48 h for cell viability determinations or for 24 h for measurements of DNA fragmentation, the levels of caspase-3, Bcl-2 and Bax, and the Bax/Bcl-2 ratio. (**A**) Cell viability measured with 3-(4,5-dimethylthiazol-2-yl)-2,5-diphenyltetrazolium bromide (MTT) assay. * *p* < 0.05 vs. the corresponding “None”. “None” corresponds to the untreated cell extract; “50”, “100”, and “150” correspond to the extracts of cells treated with 50, 100, and 150 µM DHA, respectively. (**B**) DNA fragmentation determined from the amount of oligonucleosome-bound DNA detected in the cell extracts. The description of the columns is the same as in (**A**). (**C**) Levels of capase-3, Bcl-2, and Bax in whole cell extracts determined by Western blot analysis. The description of the columns is the same as in (**A**). (**D**) The ratio of Bax/Bcl-2 determined from the Bax and Bcl-2 protein band densities. * *p* < 0.05 vs. “None”. The description of the columns is the same as in (**A**). The Bax/Bcl-2 ratio for “None” was set at 1.

To determine the effect of DHA on growth of PANC-1 cells, cell viability was measured using the MTT assay. As shown in Figure 1A, the viability of the cells treated with DHA decreased in a dose-dependent manner in contrast to that of cells not exposed to DHA. To examine whether the decreased cell viability was due to the apoptotic effect of DHA, DNA fragmentation was probed by measuring the amount of nucleosome-bound DNA in DHA-treated vs. untreated cells. As is illustrated in Figure 1B, DNA fragmentation increased in response to DHA in a dose-dependent manner. Caspase-3 exists as an inactive proenzyme, which is cleaved to the active enzyme in response to apoptosis-inducing stimuli. DHA exposure increased the level of cleaved caspase-3 (see Figure 1C), which demonstrates that DHA induces apoptosis in PANC-1 cells. DHA also increased the level of pro-apoptotic Bax and decreased the level of anti-apoptotic Bcl-2 (Figure 1C), resulting in a dose-dependent increase in the Bax/Bcl-2 ratio (Figure 1D).

### 3.2. DHA Suppresses the Activity of NF-κB in PANC-1 Cells

**Figure 2:**
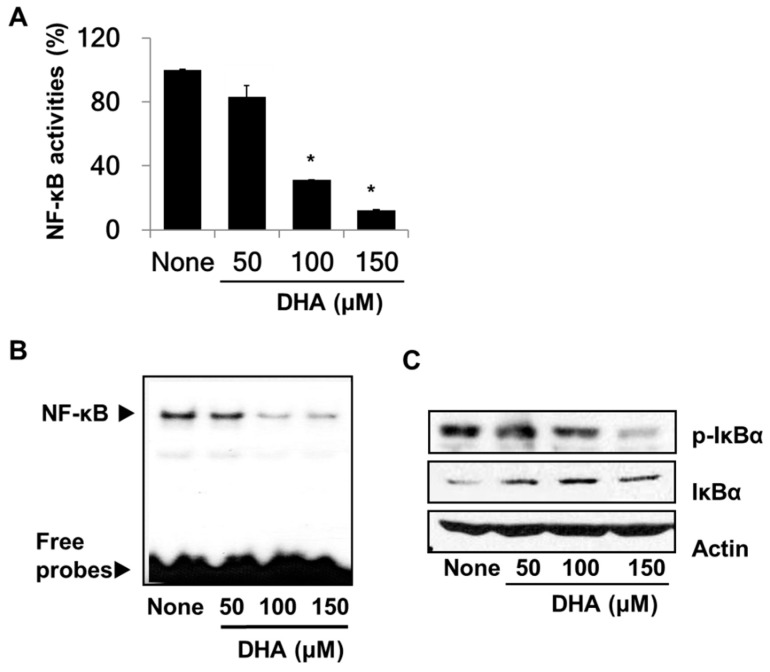
Effect of DHA on nuclear transcription factor-κB (NF-κB) activation and IκBα levels in PANC-1 cells. Cells were pre-treated with the indicated concentrations of DHA for 4 h. (**A**) DNA-binding activity of NF-κB was examined by the luciferase assay. * *p* < 0.05 vs. “None”. Column “None” corresponds to the extract from untreated cells, and columns “50”, “100”, and “150” to the extracts of cells treated with 50, 100, and 150 µM DHA, respectively. (**B**) Audioradiogram of the Electrophoretic Mobility Shift Assay (EMSA) gel on which nuclear extracts treated with a [^32^P]-oligonucleotide probe for NF-κB were chromatographed. The description of the columns is the same as in (**A**). (**C**) Western blot analysis of phospho-specific IκBα and IκBα (and the protein standard actin) present in untreated cells (column “None”), and the cells pre-treated with 50, 100, and 150 µM DHA, respectively (columns “50”, “100”, and “150”).

To demonstrate the inhibitory effect of DHA on the activity of NF-κB in PANC-1 cells, NF-κB DNA-binding activities were determined by using the luciferase reporter assay and the EMSA. Both assays showed significantly reduced DNA binding activities of NF-κB in DHA-treated cells (Figure 2A,B). Because the activity of NF-κB is regulated by IκBα, the effect of DHA on the phosphorylation of IκBα was assessed. Western blot analysis (Figure 2C) revealed less phosphorylated IκBα and more unphosphorylated IκBα in the DHA-treated cells. This result indicates that DHA inhibits NF-κB activation by inhibiting phosphorylation of IκBα in PANC-1 cells.

### 3.3. DHA Inhibits Activation of STAT3 and EGFR in PANC-1 Cells

**Figure 3:**
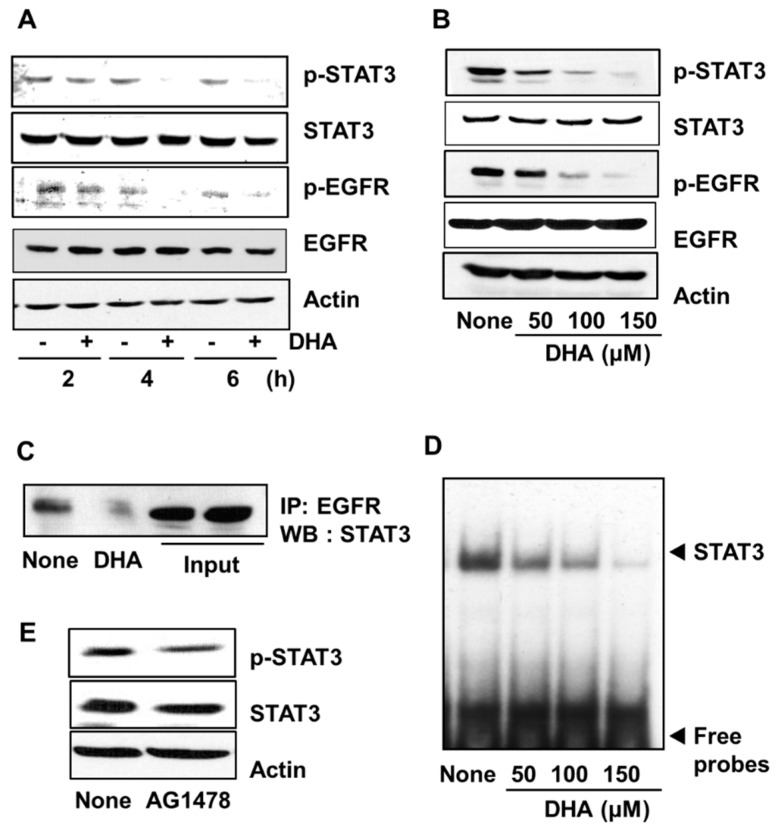
Effect of DHA on the levels of phospho (p)-STAT3, STAT3, p-EGFR, and EGFR, the interaction of EGFR and STAT3, STAT3 DNA-binding activity, and the effect of AG1478 on p-STAT3 and STAT3 levels in PANC-1 cells. Cells were treated with 150 µM DHA for various time periods (**A**) and with 50, 100, and 150 µM DHA for 4 h (**B**–**D**). The cells were treated with 10 μM tyrophostin AG1478 for 4 h (**E**). (**A**) Plot of the protein levels of p-STAT3, STAT3, p-EGFR, and EGFR (and the protein standard actin) in the cells treated with 150 µM DHA for 2, 4, and 6 h. “-” means without DHA treatment and “+” represents with DHA treatment. (**B**) Plot of the protein levels of p-STAT3, STAT3, p-EGFR, and EGFR (and the protein standard actin) in the cells treated for 4 h with the concentrations of DHA indicated. Column “None” corresponds to the extract from untreated cells, column “50”, “100”, and “150” to the extracts from cells treated with 50, 100, and 150 µM DHA, respectively. (**C**) Western blot (WB) of PANC-1 cells treated with 150 µM DHA for 4 h. The total cell lysates were immunoprecipitated (IP) with EGFR antibody and then immunoblotted (by Western blotting, WB) using STAT3 antibody. (**D**) DNA-binding activity of STAT3 in PANC-1 cells treated for 4 h with the indicated concentrations of DHA. The description of the columns is the same as in (**B**). (**E**) Plot of the protein levels of p-STAT3 and STAT3 (and the protein standard actin) in the cells treated with 10 µM AG1478 for 4 h. The column labeled “None” corresponds to the extracts from untreated cells and the column labeled “AG1478” corresponds to the extracts from cells treated with 10 µM AG1478. EGFR = epidermal growth factor receptor (EGFR); STAT3 = signal transducer and activator of transcription factor 3.

To examine the effect of DHA on STAT3 activation, PANC-1 cells were treated with 150 µM DHA for 2, 4, and 6 h. The level of phosphorylated STAT3 decreased in a time-dependent manner whereas no change in the level of total STAT3 occured (Figure 3A). Likewise, STAT3 phosphorylation decreased with increasing concentrations (50, 100, and 150 µM) of DHA used in a 4 h treatment of the PANC-1 cells (Figure 3B). To investigate whether the decreased level of phosphorylation of STAT3 is a result of decreased EGFR activation, the phosphorylation level of EGFR was determined by using Western blot analysis. In addition, the effect of DHA on the association of EGFR and STAT3 was examined by carrying out immunoprecipitation of the EGFR-STAT3 complex. Treatment of cells with DHA suppressed phosphorylation of EGFR in a dose- and time-dependent manner (Figure 3A,B). Moreover, the level of the EGFR-STAT3 complex in cells treated with 150 µM DHA for 4 h was decreased compared to that in untreated cells (Figure 3C). Next, the effect of DHA on the DNA-binding activity of STAT3 was examined by using the EMSA. As shown in Figure 3D, treatment with DHA decreased STAT3 DNA-binding activity in a dose-dependent manner (Figure 3D). Lastly, the highly potent and specific EGFR tyrosine kinase inhibitor, AG 1478 was used to reduce the amount of activated EGFR in the PANC-1 cells, and the impact on the phosphorylation of STAT3 was determined. As shown in Figure 3E, STAT3 phosphorylation was reduced. Taken together, these results demonstrate that DHA treatment reduces EGFR activation, and hence, STAT3 activation for DNA-binding.

### 3.4. DHA Disintegrates Lipid Rafts, Resulting in Exclusion of EGFR from Lipid Rafts in PANC-1 Cells

**Figure 4:**
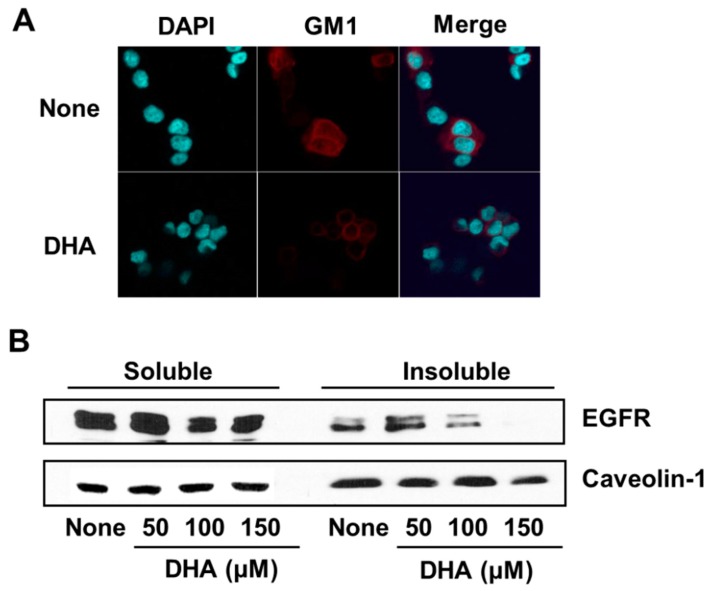
The effect of DHA on monosialotetrahexosylganglioside (GM1) and EGFR levels in the lipid rafts of PANC-1 cells. Cells were treated for 4 h with 150 µM DHA (**A**) or the indicated concentrations of DHA (**B**). (**A**) Confocal microscope images. Fixed cells were stained with Alexa-fluor 555-conjugated cholera toxin subunit B (CTB-555) to identify the GM1 of lipid rafts, and with 4′,6-diamidino-2-phenylindole (DAPI) to locate the nucleus. The row of images labeled “None” corresponds to the extract from untreated cells and that labeled “DHA” to the extracts of cells treated with 150 µM DHA. (**B**) Western blot detection of EGFR associated with the soluble vs. insoluble fractions of PANC-1 cellular membranes generated using the detergent Triton X-100. Caveolin-1 was used as a marker for the plasma membrane. The column labeled “None” corresponds to the extract from untreated cells, and the columns labeled “50”, “100”, and “150” correspond to the extracts of cells treated with 50, 100, and 150 µM DHA, respectively.

In order to determine the effect of DHA on the integrity of membrane lipid rafts in PANC-1 cells, the marker for lipid rafts, GM1, was stained with Alexa-fluor 555-conjugated cholera toxin subunit B (CTB-555) and detected using a confocal microscope. As shown in Figure 4A, GM1 is less visible in DHA-treated cells than in cells without treatment, indicating that DHA disintegrated the lipid rafts.

In addition, the amount of EGFR in the detergent-insoluble membrane fraction, in which lipid rafts are present, decreased following DHA treatment (Figure 4B). The amount of calveolin-1, as a marker of plasma membrane, was not decreased by DHA treatment in membrane fraction (Figure 4B). These results show that DHA mediates the exclusion of EGFR from lipid rafts and may attenuate EGFR signaling in PANC-1 cells.

### 3.5. DHA Decreases Expression of Cyclin D1 and Survivin in PANC-1 Cells

**Figure 5:**
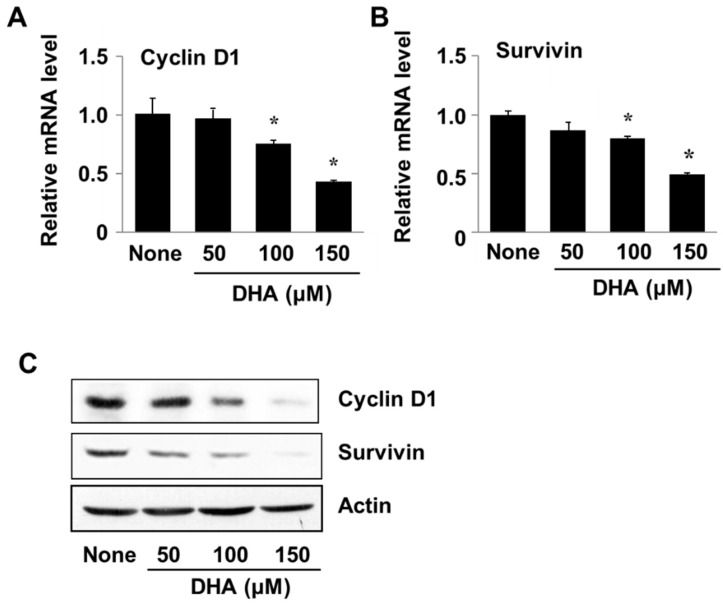
The effect of DHA on cyclin D1 and survivin gene expression in PANC-1 cells. Cells were treated with DHA for 24 h. (**A**,**B**) A plot of the relative levels of cyclin D and survivin messenger RNA (mRNA), determined by using real-time PCR analysis. * *p* < 0.05 vs. None. Column “None” corresponds to the untreated cell extract, column “50”, “100”, and “150”to the extracts of cells treated with 50, 100, and 150 µM DHA, respectively. (**C**) Western blot detection of cyclin D and survivin. The description of the columns is the same as in (**A**,**B**).

Cyclin D1 and survivin are involved in cell proliferation and survival and are regulated at the gene level by transcription factors such as STAT3 and NF-κB. To examine the effect of DHA on expression of the respective genes *CCND1* and *BIRC5* (for cyclin D1 and survivin), PANC-1 cells were treated with DHA for 24 h after which the levels of encoded mRNA and protein were determined. Cells treated with DHA showed decreased levels of the transcribed mRNAs as well as the translated proteins (Figure 5A,B). These results demonstrate that DHA induces apoptosis of PANC-1 cells by suppressing expression of the cyclin D1 and survivin genes.

## 4. Discussion

Many studies have focused on the control of aberrantly activated STAT3 in cancer cells because it plays important roles in anti-apoptotic and pro-proliferative signaling [31,32]. Recently, pre-clinical and clinical studies carried out with STAT3 inhibitors have shown that targeting STAT3 offers a promising therapeutic approach to cancer treatment [33]. Among the numerous ways to activate STAT3, phosphorylation catalyzed by EGFR, a member of ErbB family of Type 1 receptor tyrosine kinases, is the best understood [34,35]. EGFR is commonly over-expressed in pancreatic cancer cells and, thus, we chose the EGFR-STAT3 pathway as the focus our study.

We first showed that DHA induces apoptosis in PANC-1 cells. Next, we demonstrated that DHA suppresses the activation of EGFR and its association with, and activation of, STAT3. The reduction in DNA binding by STAT3 in DHA-treated cells is consistent with the loss of STAT3 transcriptional activity and thus, with increased apoptosis in response to DHA.

To gain insight into the mechanism by which DHA suppresses EGFR activation, the effect of DHA on the level of EGFR in cell membrane was examined. Whereas the level of the plasma membrane marker calveolin-1 was not affected by DHA, the level of membrane EGFR was decreased. To discover why this is the case, we investigated the effect of DHA on the association of EGFR with lipid rafts. Lipid rafts are enriched in shingolipids and cholesterol, and serve as cell membrane microdomains for the co-location of signaling proteins such as EGFR, Src, Hsp90, and Akt. [27,36]. The spingolipid GM1 is widely used as a marker of lipid rafts [37]. By measuring the response of GM1 levels to DHA using the GM1 receptor cholera toxin B for detection [38], we found that DHA decreased the GM1 levels. This result indicated that DHA alters the lipid rafts of PANC-1 cell membranes.

The alteration of lipid rafts and raft-associated proteins through the depletion or replacement of the raft component cholesterol is known to affect a number of cell signaling pathways [39,40]. Thus, DHA may alter the localization of EGFR within the lipid rafts, influencing its signaling [41,42]. To test this proposal, the effect of DHA on the level of EGFR associated with lipid rafts was examined. Because lipid rafts are known to be resistant to nonionic detergents, the membrane was separated into soluble and insoluble fractions by using Triton X-100. Measurement of the level of EGFR in these fractions showed that the EGFR level in the insoluble fraction decreased upon DHA treatment, which we showed also disrupted the lipids rafts. According to Turk et al. [43], DHA mediates increased EGFR internalization and degradation, suppressing the ability of EGFR to activate downstream signaling cascades. In this way, the treatment PANC-1 cells with DHA may reduce the integrity of the lipid rafts and induce the exclusion of EGFR. 

DHA also inhibited the activity of NF-κB in PANC-1 cells. Fan et al. [44] reported that NF-κB and STAT3 signaling pathways collaborate in cancer cells via direct interaction and by cooperatively binding at a subset of gene promoters to synergistically induce their target genes. Through investigation, DHA was shown to inhibit the expression of the genes encoding cyclin D1 and survivin, both of which are regulated by STAT3 and NF-κB. In this sense, inhibition of STAT3 by DHA may influence NF-κB activation and thus suppress target genes in PANC-1 cells. We showed that DHA inhibited the phosphorylation of IκBα, which suppressed NF-κB activation. This finding is supported by previous work which showed that DHA pretreatment of hairless mouse skin attenuated ultraviolet (UV) B-induced DNA-binding of NF-κB through the inhibition of phosphorylation of IκB kinase-α/β, phosphorylation and degradation of IκBα, and nuclear translocation of p50 and p65 [45].

Das and Das showed that treatment of neoplastic cells C6 glioma and SH-SY5Y cell lines with 100 µM DHA for 24 h showed significant loss of cell viability. They found that excess reactive oxygen species (ROS) and activated mitogen-activated protein kinase (MAPK) promoted cell death in cancer cells [46]. DHA depleted intracellular glutathione and decreased mitochondrial function, resulting in increased ROS levels in cancer cells [47,48]. In lung cancer cells, DHA induced apoptosis by increasing MAPK-phosphatase-1 (MKP-1) and down-regulating p-extracellular signal–regulated kinases (ERK) ½, and p-p38 expression [49]. In breast cancer cells, DHA significantly increased the ratio of cyclic adenosine monophosphate (cAMP)/cyclic guanosine monophosphate (cGMP) levels and promoted the expression of Toll-like receptor 4 (TLR-4) and peroxisome proliferator activated receptor (PPAR)-α, resulting in apoptosis. This study suggested a clinical development of DHA oil in breast cancer treatment [50]. DHA-induced apoptosis mediated ROS-Akt-mammalian target of rapamycin (mTOR) signaling in prostate cancer cells [51]. Taken together, DHA-induced cancer cell apoptosis may be mediated with excess ROS, activation of MAPKs, MKP-1, STAT3-NF-κB, Akt, and mTOR signaling and increased in cAMP/cGMP, TLR-4, and PPAR-α. Further studies should be performed to investigate other apoptotic mechanisms of DHA other than the STAT3-NF-κB signaling in the present experimental setting.

In the present study using PANC-1 cells, we found that DHA induced apoptosis is accompanied by increased DNA fragmentation and caspase-3 activation, and by a decreased ratio of anti-apoptotic protein Bcl-2 to pro-apoptotic protein Bax. DHA inhibited the activation of EGFR by inhibiting lipid raft formation thus excluding EGFR from lipid rafts. DHA also inhibited the activation and hence, DNA-binding activities of NF-κB and STAT3. This in turn down-regulated the expression of their targeted cyclin D1 and survivin genes, which are known to contribute to the regulation of cell growth and survival. Based on these findings we propose that dietary supplementation with DHA-rich foods may prevent or delay the development of pancreatic cancer.
